# Regulation of Eukaryotic RNAPs Activities by Phosphorylation

**DOI:** 10.3389/fmolb.2021.681865

**Published:** 2021-06-25

**Authors:** Araceli González-Jiménez, Adrián Campos, Francisco Navarro, Andrés Clemente-Blanco, Olga Calvo

**Affiliations:** ^1^ Instituto de Biología Funcional y Genómica, Consejo Superior de Investigaciones Científicas, Universidad de Salamanca, Salamanca, Spain; ^2^ Departamento de Biología Experimental-Genética, Universidad de Jaén, Jaén, Spain; ^3^ Centro de Estudios Avanzados en Aceite de Oliva y Olivar, Universidad de Jaén, Jaén, Spain

**Keywords:** phosphorylation, transcription regulation, gene expression, RNA polymerase I, RNA polymerase II, RNA polymerase III

## Abstract

Evolutionarily conserved kinases and phosphatases regulate RNA polymerase II (RNAPII) transcript synthesis by modifying the phosphorylation status of the carboxyl-terminal domain (CTD) of Rpb1, the largest subunit of RNAPII. Proper levels of Rpb1-CTD phosphorylation are required for RNA co-transcriptional processing and to coordinate transcription with other nuclear processes, such as chromatin remodeling and histone modification. Whether other RNAPII subunits are phosphorylated and influences their role in gene expression is still an unanswered question. Much less is known about RNAPI and RNAPIII phosphorylation, whose subunits do not contain functional CTDs. However, diverse studies have reported that several RNAPI and RNAPIII subunits are susceptible to phosphorylation. Some of these phosphorylation sites are distributed within subunits common to all three RNAPs whereas others are only shared between RNAPI and RNAPIII. This suggests that the activities of all RNAPs might be finely modulated by phosphorylation events and raises the idea of a tight coordination between the three RNAPs. Supporting this view, the transcription by all RNAPs is regulated by signaling pathways that sense different environmental cues to adapt a global RNA transcriptional response. This review focuses on how the phosphorylation of RNAPs might regulate their function and we comment on the regulation by phosphorylation of some key transcription factors in the case of RNAPI and RNAPIII. Finally, we discuss the existence of possible common mechanisms that could coordinate their activities.

## Introduction

The transcription of cellular RNAs is carried out by DNA-dependent RNA polymerases (RNAPs). In bacteria and archaea, only one RNAP transcribes all RNAs. In Eukarya, three RNAPs (RNAPI, -II and -III) are required for RNA transcription, except plants containing two other RNAPs (RNAPIV and -V). RNAPI synthesizes the precursor ribosomal RNA (rRNA 35S in yeast, 47S in mammals), RNAPIII produces 5S rRNA and transfer RNAs (tRNAs) and RNAPII transcribes all the protein-coding genes synthesizing messenger RNAs (mRNAs). Additionally, RNAPII and RNAPIII can synthesize other types of transcripts, such as small non-coding RNAs (ncRNAs), whose specific synthesis may differ depending on the species (Huet et al., 1985; Dieci et al., 2007). Finally, RNAPIV and RNAPV produce small interfering (siRNAs) and ncRNAs in plants (Onodera et al., 2005; Zhang et al., 2007; Haag and Pikaard, 2011; Lopez et al., 2011; Wang and Ma, 2015). All RNAPs are related at the evolutionary level, displaying common structures and functions. The minimum preserved structure of RNAPs is that of bacteria, consisting of five subunits. Archaeal RNAP has 12 subunits and eukaryotic RNAPs are complexes of 12 (RNAPII), 14 (RNAPI) and 17 (RNAPIII) subunits (Cramer et al., 2008; Werner and Grohmann, 2011; Wang and Ma, 2015; Cramer, 2019b). They all have a structurally conserved core formed by 10 subunits, with additional factors located on the polymerase complex periphery. Moreover, they all share five subunits (Rpb5, Rpb6, Rpb8, Rpb10, and Rpb12) with common functions but also with specific roles in their corresponding RNAPs (Cramer et al., 2008; Cuevas-Bermúdez et al., 2017). The structures of the three eukaryotic RNAPs, first solved in *Saccharomyces cerevisiae,* are highly conserved and their resolution has tremendously helped to understand the mechanism of transcription (Cramer et al., 2000; Armache et al., 2005; Engel et al., 2013; Fernandez-Tornero et al., 2013; Hoffmann et al., 2015; Sainsbury et al., 2015; Ramsay et al., 2020; Schier and Taatjes, 2020). The correct regulation of gene transcription depends on mechanisms that regulate the formation of large multiprotein complexes (RNAPs and their cognate factors) and their dynamics through all the transcription process. One of the most prominent mechanisms is post-translational modification (PTM) of proteins (Deribe et al., 2010), phosphorylation being the most frequent (Beltrao et al., 2013). A clear example is the dynamic phosphorylation of the carboxyl-terminal domain (CTD) of Rpb1, key for gene transcription (Buratowski, 2009; Calvo and García, 2012; Hsin and Manley, 2012; Eick and Geyer, 2013; Harlen and Churchman, 2017). Unfortunately, while most of the available data refer mainly to the phospho-regulation of transcription factors implicated in the modulation of all RNAP activities, little is known about the phosphorylation of other RNAP subunits and their implications in RNA biogenesis. Here, we have compiled all the phospho-sites identified to date for *S. cerevisiae* and human RNAPs (Supplementary Tables S1, S2). We discuss the localization and possible roles of the three RNAP subunit phosphorylations in budding yeast, as the structures of the different transcription complexes are better known in this organism. Finally, we review the possible conservation of RNAP phospho-regulation with evolution.

## RNAPII Phosphorylation

RNAPII is the best known of the eukaryotic RNA polymerases. Transcription by RNAPII is a very complex, dynamic and finely regulated process. A sophisticated network of protein–protein and protein–nucleic acid interactions is established, producing conformational and activity changes in RNAPII through the transcription cycle. Thus, a pre-initiation complex (PIC), composed basically of general transcription factors (GTFs: TFIIA, B, D, E, F, and H), Mediator and RNAPII, is assembled at the gene promoters, opening the DNA to initiate transcription (Greber and Nogales, 2019; Schier and Taatjes, 2020). Other factors acting as activators/co-activators and repressors/co-repressors can modulate the transcription activity (Ho and Shuman, 1999; Thomas and Chiang, 2006; Hahn and Young, 2011; Roeder, 2019). Subsequently, RNAPII activity is regulated by elongation and termination factors (Kwak and Lis, 2013). Because pre-mRNA maturation (capping, splicing and polyadenylation) occurs co-transcriptionally, a set of processing factors also interacts with the transcription machinery. Moreover, chromatin and histone modifiers act to facilitate and regulate the passage of RNAPII through the genes being transcribed in concert with the transcription complex. It is well known that the correct orchestration of all these processes involved in mRNA biogenesis is coordinated and fine-tuned by the phosphorylation status of the Rpb1-CTD (Perales and Bentley, 2009; Calvo and García, 2012; Hsin and Manley, 2012; Harlen and Churchman, 2017).

Functional and structural studies with *S. cerevisiae* have provided the majority of the existing knowledge about RNAPII transcription mechanisms, regulation and coordination with other cellular processes (Cramer, 2019a; Cramer, 2019b; Roeder, 2019). Recent structural data combined with functional studies have advanced our understanding of RNAPII transcription in general and that of PIC function, structure and dynamics in particular (Greber and Nogales, 2019; Schier and Taatjes, 2020). Resolution of the RNAPII structure by X-ray crystallography about 20 years ago showed that its twelve subunits are folded and assembled into four mobile modules: the *core* module, formed by the active center (Rpb1 and Rpb2) and assembly platform (Rpb3, Rpb10, Rpb11, and Rpb12); the *jaw-lobe* module, made up of Rpb1 and Rpb9; the *shelf* module containing the foot and cleft domains of Rpb1 and the lower jaw and assembly domains of Rpb5; and the *stalk* module, formed by Rpb4 and Rpb7, which in the case of *S. cerevisiae* can be dissociated from the 10-subunit core polymerase (Cramer et al., 2000; Cramer et al., 2001; Gnatt et al., 2001; Armache et al., 2003; Bushnell and Kornberg, 2003). Within these modules there are some key structural domains with basic roles in transcription, such as the active site, cleft, clamp, wall, protrusion, funnel and RNA exit channel. Movement of these regions is accompanied by binding of the GTFs with essential roles in transcription initiation. Subsequent binding of elongation factors replaces the GTFs, thus regulating further steps of the transcription cycle. How all these events take place is not fully understood, although some are explained by conformational changes of the transcription complex and/or phosphorylation of specific factors and the Rpb1-CTD (Wang et al., 2010; Larochelle et al., 2012; He et al., 2013; Sainsbury et al., 2015; He et al., 2016; Harlen and Churchman, 2017; Nogales et al., 2017; Greber and Nogales, 2019; Nogales and Greber, 2019; Patel et al., 2019; Schier and Taatjes, 2020).

The Rpb1-CTD is an unstructured and flexible domain that is crucial for the regulation of RNAPII transcription. It consists of multiple repeats of the heptapeptide sequence Tyr1-Ser2-Pro3-Thr4-Ser5-Pro6-Ser7, which is not present in other RNAPs. It is evolutionarily well conserved from protozoa to metazoa. The number of repeats ranges from 26 repetitions in *S. cerevisiae* to 52 in mammals (Corden et al., 1985; Chapman et al., 2008). Five of the seven residues are susceptible to phosphorylation: Tyr1; Ser2, -5, and -7; and Thr4 (Buratowski, 2003; Hsin et al., 2011; Hsin and Manley, 2012; Allepuz-Fuster et al., 2014; Yurko and Manley, 2018). Prior to transcription initiation, the Rpb1-CTD likely interacts with Mediator and helps to recruit it to the promoter (Kim et al., 1994; Naar et al., 2002; Robinson et al., 2016). During initiation, the CTD becomes phosphorylated by transcription-associated kinases, generating phospho-marks required for the binding of elongation and RNA processing factors, among others, and to proceed to a productive elongation phase. As this subject has been extensively reviewed (Perales and Bentley, 2009; Calvo and García, 2012; Hsin and Manley, 2012; Harlen and Churchman, 2017), we will focus on this section on the phosphorylations of other RNAPII subunits (Supplementary Table S1), although in most cases a role in transcription and/or RNA processing has not been yet stated.

**FIGURE 1:**
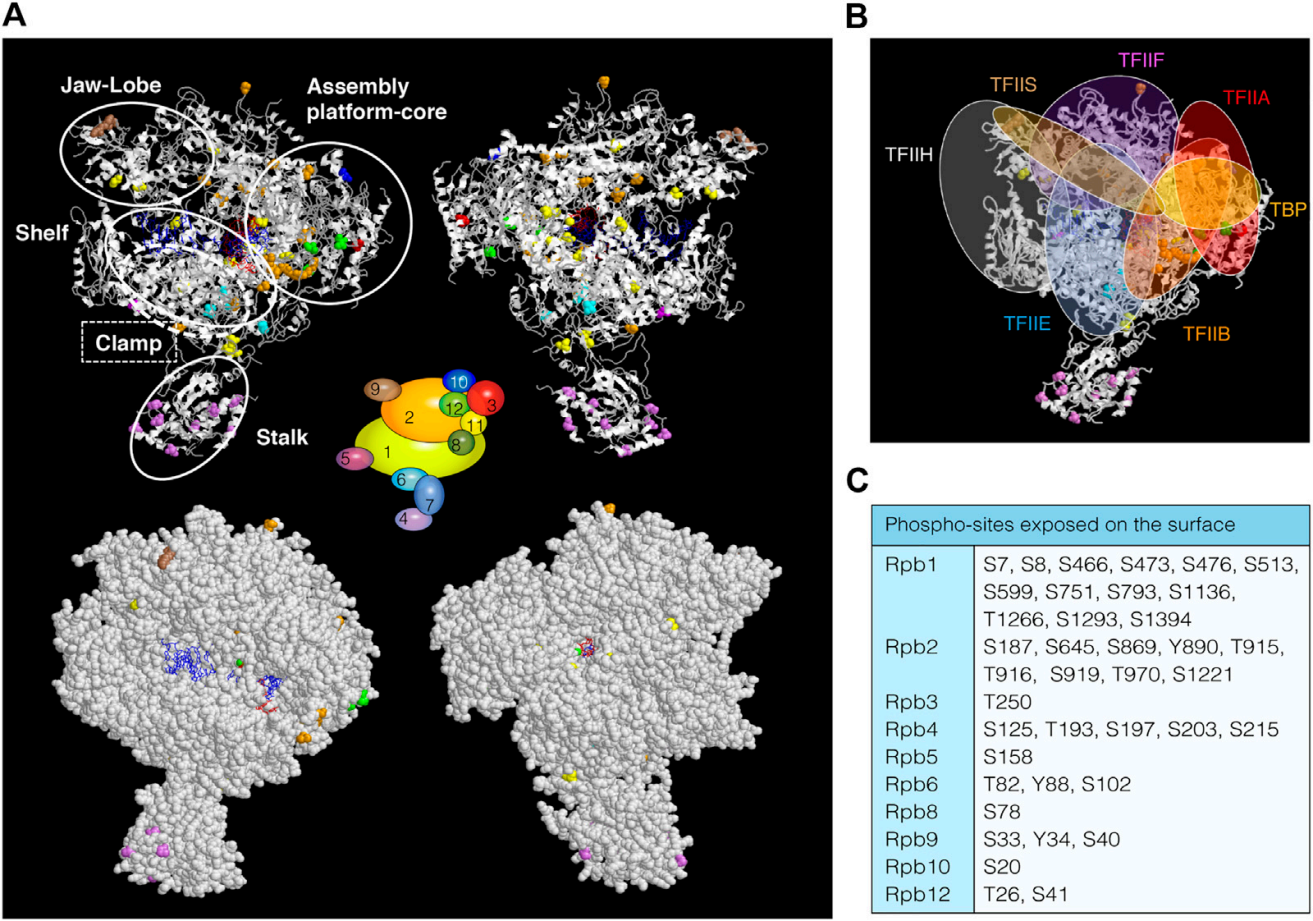
RNAPII phospho-sites. **(A)** Upper, schematic views (ribbon representation) of *Saccharomyces cerevisiae* RNAPII (PDB: 1y1w), displaying phospho-sites that have been labelled in different colours according to the 12 subunits diagram shown in the middle of the figures. RNAPII mobile modules are indicated with white open circles. DNA is represented in blue and RNA in red. Surface **(*bottom*)** views showing exposed phospho-sites. **(B)** Schematic representation of GTFs localizations according to published works (i.e., (Sainsbury et al., 2015; Schier and Taatjes, 2020)). **(C)** Table with phospho-sites exposed on the surface of RNAPII whose phosphorylation status could be important for the association/dissociation of transcription regulators.

Several phospho-proteomic studies have identified at least 75 new phospho-sites in 10 of the 12 subunits of *S. cerevisiae* RNAPII (Supplementary Table S1), 55 of them distributed along specific RNAPII subunits (Rpb1, Rpb2, Rpb3 Rpb4, and Rpb9) and 20 in shared subunits with RNAPI and RNAPIII (Rpb5, Rpb6, Rpb8, Rpb10, and Rpb12) (Albuquerque et al., 2008; Pultz et al., 2012; Swaney et al., 2013; Sostaric et al., 2018; MacGilvray et al., 2020; Lanz et al., 2021; Richard et al., 2021). However, it is unknown whether all these residues are phosphorylated *in vivo* and if they form part of a regulatory mechanism to control the biogenesis of RNAPII transcripts. Localization of these residues on the RNAPII structure (Figure 1A, upper panel) shows a broad distribution, with 50 of the 75 phospho-sites localized in structured regions. Notably, many of them correspond to defined RNAPII regions, suggesting that post-translational modifications by phosphorylation may influence how these specific regions act during the transcription steps (i.e., DNA contact, NTP addition, clamp movement, etc.). It is worth noting that 39 phospho-sites are exposed on the surface of the enzyme (Figure 1A, bottom panel, and Figure 1C). Thus, it is tempting to speculate that the phosphorylation of these residues might be important for protein–protein interaction between RNAPII and different transcriptional regulators. In fact, these exposed residues localize in regions that are described to contact the GTFs (TFIIB, TBP, TFIIA, TFIIE, TFIIF, TFIIH or TFIIS; Figure 1B), Mediator and elongation factor Spt5/4 (Cai et al., 2010; Martinez-Rucobo et al., 2011; Nogales et al., 2017; Greber and Nogales, 2019; Schier and Taatjes, 2020). Interestingly, there are three phospho-sites (S1793, T1471, and Y1473) within the Rpb1 *linker*, an unstructured region that connects the CTD to the rest of the protein, whose phosphorylation is required for Spt6 interaction with RNAPII and the re-assembly of repressive chromatin during transcription (Sdano et al., 2017).

Rpb2 phosphorylation sites lie in the external 1, protrusion, fork, wall, hybrid binding and anchor domains. In the wall, near the active site, the RNA:DNA hybrid separates and upstream DNA makes a 90° turn to exit RNAPII (Cramer et al., 2001). The protrusion is an external, positively charged domain, placed above the wall where the DNA exits from the cleft. Re-annealing of transcribed DNA occurs as it exits the enzyme, and the protrusion may participate in this process. Therefore, phosphorylation of residues lying in these domains could be involved in the separation of the RNA:DNA hybrid, the re-annealing of the transcribed DNA as it exits the enzyme and/or in the association/dissociation of transcription and processing factors (Pappas and Hampsey, 2000). Another example is the association and function of TFIIB and TFIIF during transcription initiation, factors important to position the DNA over the RNAPII active center cleft (Sainsbury et al., 2015; Greber and Nogales, 2019; Schier and Taatjes, 2020). Indeed, TFIIB interacts with the clamp, with the dock and cleft (Rpb1), and with the wall and protrusion domains (Rpb2) (Kostrewa et al., 2009; Liu et al., 2010; Sainsbury et al., 2015). TFIIF binds upstream and downstream DNA and RNAPII near the Rpb2 lobe and protrusion domains (Muhlbacher et al., 2014; Plaschka et al., 2015). Again, phosphorylation of residues in TFIIB and TFIIF binding regions could be involved in their association with the RNAPII.

Rpb4 and Rpb7 form a heterodimer known as the stalk domain, and only Rpb4 contains phosphorylation sites (Richard et al., 2021). The stalk extends from the foot domain at the base of the RNAPII enzyme and its movement helps to coordinate opening and closing of the clamp (Armache et al., 2003; Bushnell and Kornberg, 2003). It is contacted by initiation and elongation factors (Cai et al., 2010; Martinez-Rucobo et al., 2011; Li et al., 2014; Plaschka et al., 2015; Greber and Nogales, 2019; Schier and Taatjes, 2020). Rpb4 contains several phospho-sites whose phosphorylation may be important for interaction with Rpb7 and/or the 10-subunit polymerase. Accordingly, the Rpb4 S125 residue resides within a region exclusively present in *S. cerevisiae* that could regulate specific functions in this organism, such as dissociation of Rpb4/7 from the core polymerase (Sharma and Kumari, 2013; Duek et al., 2018). Moreover, exposed residues could mediate the association of different factors (Babbarwal et al., 2014; Garavis et al., 2017; Allepuz-Fuster et al., 2019; Calvo, 2020), depending on their phosphorylation status, such as TFIIE, TFIIF, Mediator (Cai et al., 2010; Sainsbury et al., 2015), Spt5/4 (Martinez-Rucobo et al., 2011; Li et al., 2014) some CTD phosphatases (Kimura et al., 2002; Allepuz-Fuster et al., 2014), and termination factors (Mitsuzawa et al., 2003; Runner et al., 2008), and thus regulate the function of Rpb4/7. Similarly to Rpb4/7, Rpb3 forms a heterodimer with Rpb11 (Cramer et al., 2001). Moreover, some phospho-sites fall in a region comprising the heterodimerization domain of Rpb3. This suggests that phosphorylation of this region might be important for the formation of the heterodimer. Rpb9 phosphorylated residues localized in the jaw and linker domains (Cramer et al., 2001) and, because TFIIH interacts with RNAPII near the Rpb9 jaw, we could speculate that modification of these residues could be functionally linked to this factor (Muhlbacher et al., 2014; Plaschka et al., 2015).

The subunits shared by the three RNAPs (Rpb5, Rpb6, Rpb8, Rpb10, and Rpb12) are also phospho-proteins (Supplementary Table S1) (Albuquerque et al., 2008; Swaney et al., 2013; Sostaric et al., 2018; MacGilvray et al., 2020; Lanz et al., 2021). For instance, Rpb5 and Rpb6 contain phospho-sites (S158, and Y88 and T82, respectively) localized in regions important for Rpb5 and Rpb6 assembly to RNAPII (Cramer et al., 2001; Tan et al., 2003; Zaros et al., 2007).

Modification by phosphorylation of some residues of RNAPII subunits could be important not only for the association of different factors along the transcription cycle but also for exchange of factors occupying the same or close surfaces on RNAPII. This is the case of initiation and elongation factors that compete during the transcription cycle for binding to the polymerase complex, for instance TFIIE and Spt5 (Li et al., 2014). How these mutually exclusive interactions of the transcription factors with RNAPII are regulated without affecting the efficiency of all the transcription steps (initiation, pausing and elongation) remains to be understood. Recently, it has been shown that the Rpb1-CTD undergoes liquid-phase separation, which could explain the association of initiation and elongation factors (Boehning et al., 2018; Cramer, 2019b). First, a dynamic condensate is formed near the promoter during initiation that contains a non-phosphorylated RNAPII and initiation factors. This condensate facilitates transcription initiation, RNA synthesis and Rpb1-CTD phosphorylation. Second, a transient condensate containing phosphorylated RNAPII and elongation factors is produced and maintained until RNAPII reaches the end of the genes, where RNAPII is dephosphorylated, recycled and transferred to the first condensate. As the transfer of RNAPII from one condensate to another is controlled by CTD phosphorylation, it is possible that this mechanism might be crucial for optimal transcriptional regulation (Boehning et al., 2018; Guo et al., 2019; Peng et al., 2020). However, we cannot rule out that the exchange of factors during the initiation/elongation transition could be regulated by the phosphorylation of other RNAPII subunits and/or even that the transfer of RNAPII between both condensates could require the post-translational modification of additional subunits.

Finally, the high sequence conservation within the RNAPII core between yeast and humans suggests similar mechanisms of RNA synthesis. However, sequences are more divergent toward the exterior/surface residues, suggesting that biochemically distinct interfaces interact with different factors (Cramer et al., 2001; He et al., 2013; He et al., 2016; Nogales et al., 2017; Schier and Taatjes, 2020). Accordingly, phospho-sites localized in the surface of RNAPII may contribute to the association/dissociation of species-specific factors.

## RNAPI Phosphorylation

**FIGURE 2:**
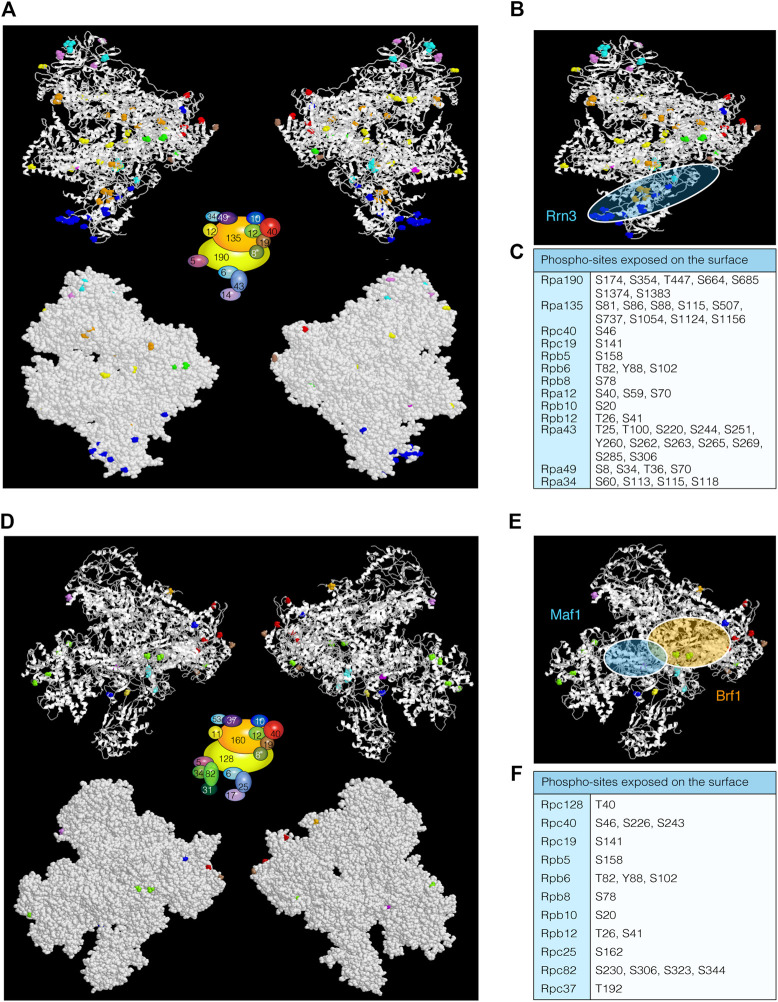
RNAPI and RNAPIII phospho-sites. **(A)** Ribbon **(*upper*)** and surface **(*bottom*)** schematic views of RNAPI (PDB: 4c3h) from *Saccharomyces cerevisiae*, displaying phospho-sites labelled in different colours according to the subunit diagram shown in the middle of the figures. **(B)** Schematic representation of Rrn3 localization (Torreira et al., 2017). **(C)** Table with phospho-sites exposed on the surface of RNAPI. **(D)** Ribbon **(*upper*)** and surface **(*bottom*)** representations of RNAPIII (PDB: 5fj9) displaying coloured phospho-sites. **(E)** Schematic representation of Maf1 and Brf1 associations with RNAPIII (Vorlander et al., 2020a; Vorlander et al., 2020b). **(F)** Table with RNAPIII phospho-sites exposed on the surface. As in the case of RNAPII, these residues could be important for the interaction with transcription regulators.

Initially, 15 phospho-sites were identified in *S. cerevisiae* distributed to five of the 14 subunits. Mutation of 13 of these phospho-sites indicated that most are non-essential PTMs, suggesting that they might contribute to non-essential RNAPI functions. Only one residue, Rpa190-S685, was suggested to play a role in rRNA cleavage/elongation or termination (Gerber et al., 2008). To date, 115 site-specific phosphorylations have been identified, mostly in phospho-proteomic studies, distributed along all the 14 RNAPI subunits (Ficarro et al., 2002; Albuquerque et al., 2008; Holt et al., 2009; Soulard et al., 2010; Pultz et al., 2012; Swaney et al., 2013; Sostaric et al., 2018; MacGilvray et al., 2020; Lanz et al., 2021). We have compiled all these sites in Supplementary Table S1. In summary, 81 sites reside in specific subunits and 34 are shared: 20 with RNAPII and RNAPIII and 14 with RNAPIII. Among these 81 phospho-sites, 63 are localized in regions of solved structure (Figure 2A, upper panel). Remarkably, 49 sites are exposed on the surface of the enzyme (Figure 2A, bottom panel, and Figure 2C), which again suggests a role for the association of RNAPI with transcription regulators, for instance Rrn3 (Figure 2B) (Torreira et al., 2017).

One of the first observations implicating protein phosphorylation in regulating RNAPI activity was the discovery that Fcp1, a Rpb1-CTD phosphatase, interacted with the RNAPI transcription machinery and was essential for rDNA efficient transcription, probably by facilitating RNAPI dephosphorylation and chain elongation during rRNA synthesis (Fath et al., 2004). Later, it was documented that the Rpa43 subunit was phosphorylated in several specific residues (S208, S220, S262, S263, S285) (Gerber et al., 2008). This subunit, together with Rpa14, forms the stalk domain and creates a platform for binding initiation factors and newly synthesized RNA (Fernandez-Tornero et al., 2013; Torreira et al., 2017). This stalk domain is required for RNAPI homodimerization and transcription inactivation (Torreira et al., 2017). However, it is unknown if Rpa43 phosphorylation levels play a role in this process. Nonetheless, it was reported that Cdc14 dephosphorylates Rpa43 in mitosis to exclude it from the nucleolus, thereby restraining rDNA transcription and facilitating condensin loading, an essential step for correct segregation of the nucleolus (Clemente-Blanco et al., 2009).

Rpa43 interacts with Rrn3 (Milkereit and Tschochner, 1998; Moorefield et al., 2000), a crucial RNAPI factor whose phosphorylation has been implicated in the regulation of the holoenzyme, after activation of growth factor signaling pathways that connect nutrient availability and rDNA production. Rrn3 is the yeast homologue of the mammalian growth-dependent rRNA synthesis factor TIF-IA (Grummt and Voit, 2010). This interaction depends on the phosphorylation of RNAPI and on Rrn3-P/TIF-IA association, and is essential to establish a competent transcriptional initiation complex (Fath et al., 2001; Cavanaugh et al., 2002; Torreira et al., 2017). Interestingly, in mice, casein kinase 2 (C1K2) has been implicated in Rrn3/TIF-IA phosphorylation at S170/172 to trigger its release from the RNAPI complex after transcription initiation, a prerequisite for transcription elongation (Bierhoff et al., 2008). This suggests that Rrn3/TIF-IA is subjected to a complex phospho-code that regulates its interaction with the RNAPI holoenzyme during ribosome biogenesis. Importantly, human RNAPI activity is also controlled in response to different types of environmental stresses throughout the phosphorylation of Rrn3/TIF-IA. Under glucose restriction, Rrn3/TIF-IA phosphorylation by the AMPK kinase prevents the assembly of a functional PIC (Hoppe et al., 2009). On the other hand, Rrn3/TIF-IA phosphorylation by the JNK kinase in mice restrains its interaction with RNAPI in response to oxidative stress, thus abrogating the formation of new PICs (Mayer et al., 2005).

## RNAPIII Phosphorylation

In terms of structural composition, RNAPIII is the largest eukaryotic RNA polymerase complex in mass and molecular conformation (Vannini and Cramer, 2012). It is formed by 17 subunits, 10 of which are unique to RNAPIII. Novel phospho-proteomic studies have shed light on the post-transcriptional phospho-mapping of multiple RNAPIII subunits (Albuquerque et al., 2008; Holt et al., 2009; Swaney et al., 2013; MacGilvray et al., 2020; Lanz et al., 2021). Fifteen of the 17 subunits are phosphorylated in both yeast and humans (Supplementary Table S1, S2). In the case of *S. cerevisiae*, there are 76 phospho-sites, 42 of them localized in specific subunits. Only 28 residues are localized in regions of known structure (Figure 2D) and 19 are exposed on the surface of the polymerase complex (Figure 2D, bottom, and Figure 2F). Three specific residues of Rpc53 (S224, T228 and T232) are of known function (see below) (Lee et al., 2012). Therefore, it is intuitive to think that RNAPIII activity might also be highly regulated by phosphorylation events, as described for RNAPI and RNAPII complexes. Interestingly, in *S. cerevisiae* only two phospho-sites in the two largest subunits have been shown to be phosphorylated, whereas 32 phospho-sites distributed along these subunits have been identified in humans (Supplementary Table S2). This observation suggests that regulation of RNAPIII activity by phosphorylation could be species specific.

Probably the best-known regulator of RNAPIII is the repressor Maf1 (Willis and Moir, 2018; Vorlander et al., 2020a), whose activity is controlled by its phosphorylation at multiple sites by protein kinase A, the rapamycin-sensitive TOR kinase (TORC1) and the TORC1-regulated kinase Sch9. Phosphorylation of Maf1 by these kinases leads to changes in its subcellular localization, a mechanism that ensures the accurate activation/repression of RNAPIII (Moir et al., 2006; Lee et al., 2009; Wei and Zheng, 2009; Willis and Moir, 2018). Additionally, casein kinase 2 (CK2) phosphorylation of Maf1 in favorable growth conditions releases this protein from the RNAPIII complex bound to genes for tRNAs, thus activating their transcription (Graczyk et al., 2011). Maf1 regulation also depends on protein phosphatases. It has been postulated that in response to nutrient starvation, poor carbon sources or several cellular stresses, Maf1 is dephosphorylated in a PP4/PP2A-dependent manner and translocated to the nucleus, thus repressing RNAPIII activity (Oler and Cairns, 2012; Ahn et al., 2019). Interestingly, nuclear localization of Maf1 is not enough to completely inhibit RNAPIII activity, suggesting the existence of alternative mechanisms that co-regulate RNAPIII transcription under these conditions (Huber et al., 2009). In agreement with this observation, recent studies have demonstrated that the RNAPIII subunit Rpc53 is also subjected to a phosphorylation switch in response to nutrient limitation and other types of cellular stress. Rpc53 phosphorylation by the two conserved kinases Kns1 and Mck1 modifies the ability of RNAPIII to interact with the DNA molecule, thus avoiding recycling rounds of transcription and allowing dephosphorylated Maf1 to join and inhibit RNAPIII activity (Lee et al., 2012). Another component of RNAPIII controlled by phosphorylation is its Rpc82 subunit, whose concomitant phosphorylation with the TFIIIB subunit Bdp1 by the Sch9 and CK2 kinases opposes Maf1-mediated transcriptional repression (Lee et al., 2015). Finally, it has been reported that the TATA-binding protein (TBP) is also a preferred substrate of CK2 *in vitro*, which suggests a new mechanism to regulate RNAPIII transcription by phosphorylation *in vivo* (Ghavidel and Schultz, 1997).

It is important to remark that RNAPIII transcription is regulated in response to environmental cues and during the different stages encompassed in the cell cycle. It has been reported that tRNA levels fluctuate during the cell cycle in a process controlled by the Cdk1/Clb5 kinase complex, boosting tRNA expression during the S phase. This is attained by the cycling phosphorylation of Bdp1, an event that triggers the recruitment of TFIIIC to the genes for tRNAs, stimulates interaction between TFIIIB and TFIIIC and enhances RNAPIII activity (Herrera et al., 2018). However, the physiological significance of cell cycle regulation of RNAPIII transcription remains to be elucidated and undoubtedly will be a fascinating question for the future.

## Coordination of RNAP Activities: Adapting Gene Expression to Environmental Conditions

RNAP activities are essential for cellular viability and a limiting step in regulating gene expression. All RNAPs respond to growth cell conditions and nutrient availability. In actively growing cells, the majority of the transcriptional output is due to RNAPI and RNAPIII activities, which are required for the synthesis of ribosomes. The activity of RNAPII is also essential because it transcribes all ribosomal protein genes and genes encoding factors required for ribosome assembly (Ribi regulon) (Warner, 1999; de la Cruz et al., 2018). How eukaryotic RNAPs are regulated has been extensively studied and is still a field of great interest. However, less is known about the mechanisms coordinating and communicating with the three RNAP machineries to adapt cell growth to environmental conditions. Factors that coordinate at least the function of two RNAPs have been identified. For instance, Spt4/5, Paf1C and Ccr4 regulate both RNAPI and RNAPII transcription (Zhang et al., 2010; Anderson et al., 2011; Hartzog and Fu, 2013; Laribee et al., 2015). Similarly, TFIIS and Sub1 influence RNAPII and RNAPIII (Guglielmi et al., 2007; Ghavi-Helm et al., 2008; Carriere et al., 2012; Garcia et al., 2012; Garavis et al., 2017; Calvo, 2018). Recently, the yeast prefoldin-like Bud27 has been shown to be a regulator of the three RNAPs, most likely via its association with a common subunit, Rpb5 (Martinez-Fernandez et al., 2020). One possibility is that RNAPs might be coordinated through regulation of the phosphorylation state of their shared subunits. In support of this hypothesis, Rpb5, Rpb6, Rpb8, Rpb10, and Rpb12 contain phospho-sites (Albuquerque et al., 2008; Swaney et al., 2013; Sostaric et al., 2018; MacGilvray et al., 2020) (Supplementary Table S1).

TOR serine/threonine kinases play an essential role in controlling many aspects of living cells, such as growth, proliferation and survival in response to nutrients (Loewith and Hall, 2011; Kim and Guan, 2019; Laribee and Weisman, 2020). Initially, it was reported that TOR proteins only localized in the cytoplasm, with a crucial role in regulating protein synthesis (Barbet et al., 1996; Gingras et al., 2004). We currently know that TOR and its associated proteins also localize in the nucleus, where they regulate gene expression to guarantee the appropriate ribogenesis (Tsang and Zheng, 2007; Laribee, 2018; Laribee and Weisman, 2020). When *S. cerevisiae* is grown under nutrient-replete conditions, Tor1 localizes in both the cytoplasm and the nucleus. In the nucleus, Tor1 and Kog1 (the Raptor subunit in *S. cerevisiae*) bind to the 35S (RNAPI) and 5S (RNAPIII) promoters. However, after starvation or rapamycin treatment, they are removed from these regions, thus inhibiting transcription (Li et al., 2006). In mammals, mTOR and Raptor also interact with the RNAPIII factor TFIIIC to induce 5S and tRNA transcription (Kantidakis et al., 2010). This and other evidence suggest that TORC1 complexes are RNAPI and RNAPIII regulators and likely coordinators of these two RNAP activities. Whether any of the RNAPI or RNAPIII subunits are phosphorylated by TORC1 is unknown. Nevertheless, RNAPIII transcription is also activated by TORC1 via phosphorylation of Maf1 in yeasts (Huber et al., 2009; Lee et al., 2009) and mammals (Kantidakis et al., 2010; Michels et al., 2010). Whereas mTOR and Raptor contribute to RNAPII transcription regulation of a number of genes in mammals (Cunningham et al., 2007; Chaveroux et al., 2013; Laribee, 2018), in budding yeast only the *HMO1* gene is known to be directly activated by Tor1 (Panday et al., 2017). Hmo1 activates the transcription of genes regulated by TORC1, including RP, 5S and 35S genes (Gadal et al., 2002; Hall et al., 2006). Both Tor1 and Hmo1 bind to the *HMO1* promoter, facilitating its transcription. After rapamycin treatment or DNA damage, Tor1 and Hmo1 are released, thus inhibiting transcription (Panday et al., 2017). Interestingly, promoter binding by the Tor1 kinase is a prerequisite for transcription inhibition, which suggests that Tor1 may phosphorylate a specific target to repress transcription in response to stress conditions. One of these targets might be Paf1C, whose activity is needed to attenuate RNAPI transcription after TORC1 inhibition (Zhang et al., 2010). Similarly, Ccr4 couples nutrient signaling through TORC1 with Rrn3-RNAPI transcription inhibition (Laribee et al., 2015). It would be reasonable to think that Tor1 kinases could also phosphorylate RNAPs, maybe common subunits, to coordinate and modulate their activities in response to environmental conditions.
