# Supplementary material for: Regulation of Eukaryotic RNAPs Activities by Phosphorylation
Source: Front Mol Biosci. 2021 Jun 25;8:681865. doi: 10.3389/fmolb.2021.681865 (PMC8268151; doi:10.3389/fmolb.2021.681865)
Supplement: Supplementary file 1 [file Table1.pdf]

RNAP II			RNAP I			RNAP III		
**Rpb1**	S	7, 8, 449, 466, 473, 476, 513, 599, 751, 793, 1136, 1247, 1293, 1449, 1493, 1521, 1532	**Rpa190**	S	147, 174, 287, 291, 297, 354, 664, 685, 889, 1358, 1359, 1360,1362, 1374, 1383, 1413, 1415, 1417, 1431, 1636	**Rpc160**	T	9
	T	191, 1266, 1394, 1471		T	150, 447, 1353, 1651			
	Y	1473		Y	149			
**Rpb2**	S	2, 7, 156, 187, 218, 493, 645, 764, 869, 919, 982, 1045, 1145, 1221	**Rpa135**	S	81, 86, 88, 115, 507, 712, 737, 1054, 1085, 1116, 1122, 1124, 1145, 1156	**Rpc128**	T	40
	T	500, 915, 916, 970, 971		T	746			
	Y	890						
**Rpb3**	S	2	**Rpc40**	S	2, 17, 24, 46, 220, 226, 243			
	T	250		T	15, 16			
**Rpb11**	-		**Rpc19**	S	62, 141			
				T	2, 15, 33			
**Rpb9**	S	33, 40	**Rpa12**	S	40, 58, 59, 70	**Rpc11**	-	
	Y	34						
**Rpb4**	S	53, 125, 197, 203, 215	**Rpa14**	S	113, 121	**Rpc17**	S	84
	T	134, 144, 193		T	120, 133			
**Rpb7**	-		**Rpa43**	S	2, 208, 220, 244, 251, 262, 263, 265, 269, 285, 306	**Rpc25**	S	162
				T	25, 100			
				Y	260			
			**Rpa34**	S	10, 12, 14, 60, 113, 115, 118, 140, 205	**Rpc37**	S	2, 52, 206
				Y	8		T	18, 61, 192
			**Rpa49**	S	8, 34, 70, 117, 151, 157, 169	**Rpc53**	S	12, 18, 27, 119, 122, 137, 138, 178, 181, 182, 224, 234, 246, 270
				T	36, 155		T	130, 228, 232, 347
						**Rpc82**	S	230, 306, 323, 344, 392, 394, 399, 421
							T	27, 430
						**Rpc34**	-	
						**Rpc31**	S	13, 189
							T	101, 190

Common RNAPs subunits			
	**Rpb5**	S	158
	**Rpb6**	S	2, 24, 102
		T	28, 41, 50, 82
		Y	29, 88
	**Rpb8**	S	73, 74, 78
		T	68
	**Rpb10**	S	20
	**Rpb12**	S	2, 20, 41
		T	19, 26

Table S1. Phospho-sites in *Saccharomyces cerevisiae* RNAPs. Rpb1-CTD phosphorylation sites are not included. Rpb5, 6, 8, 10 and 12 (in yellow boxes) are subunits shared by all three RNAPs. Rpc40 and 19 (in orange boxes) are common to RNAPI and III. S, serine; T, threonine; and Y, tyrosine. Numbers denote the corresponding amino acid of each subunit.
