# Supplementary material for: Regulation of Eukaryotic RNAPs Activities by Phosphorylation
Source: Front Mol Biosci. 2021 Jun 25;8:681865. doi: 10.3389/fmolb.2021.681865 (PMC8268151; doi:10.3389/fmolb.2021.681865)
Supplement: Supplementary file 2 [file Table2.pdf]

RNAP II		
Rpb1	S	8, 27,35, 121, 217, 269, 530, 607, 768, 772, 777
	T	37, 46, 47, 176, 368, 543, 549, 587, 608, 698, 732, 736, 1406, 1415, 1424, 1525, 1540
	Y	145, 618, 699, 763, 1383
Rpb2	S	75, 106, 354, 467, 487, 763, 937, 1012, 1016, 1147
	T	147, 218, 359, 437, 586, 870, 872
	Y	125, 215, 217, 689, 724, 736, 752, 845, 1018, 1020
Rpb3	S	124, 206, 209, 216, 235, 257
	T	12
	Y	142, 208, 220
Rpb11	T	41
Rpb9	S	51, 73
	Y	37, 54
Rpb4	S	18
	Y	67
Rpb7	S	6
	Y	3,17

RNAP I		
Rpa1	S	176, 240, 934, 1042, 1058, 1091, 1093, 1185, 1256, 1386, 1413, 1429, 1489, 1546, 1695
	T	357, 359, 360, 1373, 1487, 1580
	Y	839, 941, 1092, 1125, 1554
Rpa2	S	1051
	Y	136, 971, 998, 999, 1118
Rpa12		-
Rpa14		-
Rpa43	S	6,60, 242, 297, 299, 300, 304, 316, 328
	T	215, 254, 322
	Y	238, 302
Rpa34	S	27, 66, 124, 126, 128, 136, 163, 172, 205, 285, 309, 359, 459, 488, 490
	T	159, 171, 276, 282, 287, 363, 456, 475
	Y	80
Rpa49	S	8, 35, 49, 163, 230, 265, 296, 385, 390
	T	64, 144, 145
	Y	131

RNAP I and RNAP III		
Rpac1	S	4, 34, 157, 226, 258, 284
	T	282
	Y	33, 36, 124
Rpac2	S	12
	T	16, 25, 35

RNAP III		
Rpc1	S	24, 681, 977, 1181
	T	525, 593, 596, 948, 949, 1046, 1049, 1238, 1244, 1247, 1251, 1304
	Y	53, 434, 714
Rpc2	S	202, 206, 680, 816, 872, 873
	T	12, 304, 707, 722, 1036
	Y	710, 714
Rpc10	S	24
	T	27,35
	Y	30
Rpc9	S	37, 62
	T	46, 56
	Y	47
Rpc8		-
Rpc5	S	36, 161, 162, 192, 503, 522, 528, 544, 603
	T	236
	Y	224, 434
Rpc4	S	2, 42, 105, 107, 130, 250, 386
	T	21, 36, 59
Rpc3	S	33, 145, 194, 204, 205, 296, 329
	T	138, 147, 412, 501
	Y	301, 334
Rpc6	S	59, 79
	Y	150
Rpc7	S	157
	T	67, 133, 136
	Y	73

Common RNAPs subunits			
	RPABC1	T	29, 56, 59, 111
	RPABC2	S	2, 41
	RPABC3	T	78, 104
		Y	65, 75, 90, 118, 142
	RPABC5	Y	62
	RPABC4		-

Table S2. Phospho-sites in humans RNAPs. Rpb1-CTD phosphorylation sites are not included. RPABC1, 2, 3, 4 and 5 (in yellow boxes) are subunits shared by all three RNAPs. Rpac1 and Rpac2 (in orange boxes) are common to RNAPI and III. S, serine; T, threonine; and Y, tyrosine. Numbers denote the corresponding amino acid of each subunit.
